# Impact of a clinical program using weekly Short Message Service (SMS) on antiretroviral therapy adherence support in South Africa: a retrospective cohort study

**DOI:** 10.1186/s12911-017-0413-9

**Published:** 2017-02-20

**Authors:** Nathan Georgette, Mark J. Siedner, Carter R. Petty, Brian C. Zanoni, Stephen Carpenter, Jessica E. Haberer

**Affiliations:** 1 Harvard Medical School, Boston, MA USA; 2 Department of Medicine/Global Health, Massachusetts General Hospital, Boston, MA USA; 3 Mbarara University of Science and Technology, Mbarara, Uganda; 4 Boston Children’s Hospital, Boston, MA USA; 5 Don McKenzie Hospital, Botha’s Hill, South Africa

**Keywords:** HIV, Antiretroviral therapy, South Africa, SMS program, Adherence, Differentiated care, Program evaluation, Implementation research

## Abstract

**Background:**

In randomized controlled trials, short message service (SMS) programs have improved adherence to HIV antiretroviral therapy (ART). In response, the World Health Organization recommended use of SMS programs to support ART. However, there is limited data on real-world implementations of SMS programs.

**Methods:**

We conducted a retrospective cohort study of an SMS program to improve ART adherence in a government-run HIV clinic in rural South Africa. We analyzed data from all adult patients who 1) enrolled at the clinic before the observation period (July 2013 through June 2014), 2) had ≥1 ART prescriptions in the observation period, and 3) had data on phone number availability (*N* = 2255). Our main outcome measure was prescription coverage, defined as the presence of a valid ART prescription for each day observed. We fit generalized linear mixed models adjusted for pre-program prescription coverage, demographics, and ART duration, dosing, and regimen.

**Results:**

Exposure to the SMS program was independently associated with greater prescription coverage (AOR = 1.23, 95% CI 1.13–1.34, *P* < 0.001) compared with non-exposure, although the absolute increase in prescription coverage was small (4.7 days of ART prescription coverage per average patient per year). Among a subset of patients (*n* = 725) whose pre-program prescription coverage was <100%, the corresponding mean expected absolute increase in prescription coverage was 8.2 days per year.

**Conclusions:**

Our primary finding was that an SMS reminder program implemented in routine clinical care was associated with a small increase in prescription coverage of uncertain clinical significance.

## Background

Successful antiretroviral therapy (ART) for HIV depends on sustained, high adherence [1]. However, adherence remains a significant challenge for many [2]. Numerous barriers to adherence exist, which can be broadly categorized as individual (e.g., comorbid psychiatric conditions and forgetfulness), social (e.g., isolation and stigma), and systemic (e.g., stock-outs and inadequate counseling) [3]. Moreover, persistent adherence requires retention in care, which has also been identified as challenging [4]. Indeed, one qualitative study in sub-Saharan Africa identified missed appointments as triggers of a cascade of disengagement from care [5]. In the past several years, considerable interest has developed in using short message service (SMS), or text messaging, to address forgetfulness; others have also indicated the potential for SMS to improve the connection between patient and clinic, along with social isolation [6, 7]. SMS interventions generally fall into the following categories: daily dose reminders [8, 9], appointment reminders [10, 11], and weekly messaging aimed at providing information, adherence support, or both [9, 12–14].

Several randomized controlled trials (RCTs) have investigated the adherence benefits of weekly SMS programs. They have shown a mix of positive and null findings in terms of effect on adherence and viral suppression [12, 13, 15]. However, meta-analyses have shown an overall positive impact of SMS programs on adherence. A network meta-analysis of fourteen RCTs on adherence interventions throughout Africa found that weekly SMS significantly improved self-reported adherence and rates of viral suppression, although evidence was more limited for the latter finding [16]. Another meta-analysis of similar RCTs globally found that SMS programs could significantly improve adherence, and that weekly messaging was superior to daily [17].

The World Health Organization has recommended mobile phone text messages as an adherence support intervention for people on ART [18]. However, to our knowledge all previous publications report on SMS interventions implemented as part of research studies. Research studies typically provide significant resources to clinical infrastructure and participants directly, such as mobile phones [9], and intensive follow-up may alter behavior compared to routine clinical care. Moreover, it is difficult to assess an SMS program’s impact on adherence in the context of payments or other incentives provided for each study visit attended [9, 19]. Thus, the primary motivation of this work was to assess the implementation of an SMS program to determine whether the findings of the RCTs are borne out in a realistic clinical setting.

In this study, we evaluated a clinical program of weekly SMS reminders at a government-run HIV clinic near Durban, South Africa. Using a retrospective cohort design, we analyzed routine data collected at the clinic to determine ART prescription coverage, comparing those who were or were not sent SMS reminders.

## Methods

### Study population and clinic

This study was conducted at the Ethembeni HIV Clinic, located in a rural area approximately 40 km from Durban, South Africa. Ethembeni is a government-run, hospital-based HIV clinic with an on-site pharmacy, which provides free ART. The adult clinic population is approximately 67% female, has a median age of 37 years, and nearly all speak isiZulu as their first language.

Patients at Ethembeni receive prescriptions of varying lengths (e.g., one, two, three, or six months of ART at a time), depending on clinical needs. All prescribed medications are picked up at the on-site pharmacy. Patients with a prescription for three or fewer months generally receive all prescribed pills during the prescribing visit. Patients with a six-month prescription generally receive three months of pills at a time.

### SMS adherence program

The SMS adherence program was developed by clinic staff in collaboration with Sawubona Health (Malden, MA), a US-based non-governmental organization. Clinic staff and Sawubona Health volunteers manually entered mobile phone numbers from paper charts of adult patients into the SMS program database over two discrete periods (June-July 2012 and the month of June 2013). Notably, the clinic routinely asked each patient for a contact phone number at clinic enrollment, which was an average of 3.3 (SD 1.5) years prior to the SMS program launch date. Patients were not re-approached by staff to add a phone number for capture into the SMS program database due to program resource limitations.

The SMS program started on September 9, 2013 and is ongoing. All SMS are sent free of charge to patients; the program costs approximately 1 USD per patient per year to run. The program is opt-out; the first SMS explaining the program was sent to each adult patient with a recorded mobile phone number. This introductory SMS also informs patients how to opt-out, which can be done at any time via a reply SMS. SMS are sent to all subscribed patients each week by rotating through the following message (in isiZulu):

*Hi (First name), this is your clinic. Remember to take your pills on schedule in order to (* ***One of*** *: be strong* ***or*** *live a long life* ***or*** *feel well). Thank you.*

Starting on January 13, 2014, the program server began receiving delivery status reports of confirmed receipt of the SMS on the patient’s registered phone number. Further details on the SMS program can be found in a separate interview-based evaluation study [20].

### Data sources

Data for this analysis was extracted from routinely collected pharmacy and medical record data, as well as the SMS program data. We utilized data from November 4, 2012 through June 26, 2014, the time period for which prescription data were available. The dates, duration, and medications of each prescription were extracted for all patients at the clinic during this period. The electronic medical record extraction included age, gender, ART start date, and date of transfer out (if applicable). The SMS program data included the availability of a valid mobile phone number, date the phone number was available, date of opt-out (if applicable), and delivery status reports (from January 13 through June 26, 2014). The datasets were de-identified except for dates and collated by linking each patient to a unique study identifier (ID). An indicator variable was created which was set to one for patients with any missing values for any non-prescription covariates, and set to zero otherwise. Missing values for non-prescription covariates were replaced with the mean of all corresponding available values in the cohort.

### Statistical methods

**Fig. 1:**
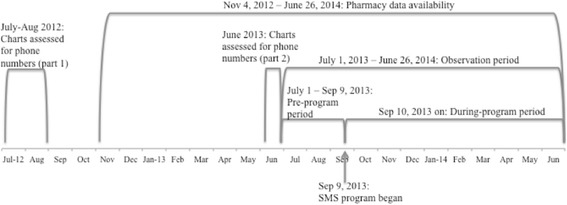
Key dates used to define the analysis periods

Figure 1 presents a visual timeline of important time periods related to this analysis. All patients satisfying the following criteria were included in the study cohort:

1. 18 years or older;
2. At least one ART prescription during the time for which both pharmacy and mobile phone data were available – the “observation period” (July 1, 2013 to June 26, 2014);
3. Enrolled at the clinic on or before July 1, 2013;
4. Phone number availability assessed prior to the observation period.

Our primary exposure of interest was inclusion in the SMS program. Clinic patients were assigned to one of the following analysis groups based on whether data from their chart had been captured into the SMS program database and on potential exposure to the SMS program (i.e., those who were and were not sent messages) via an intention-to-treat approach. That is, group assignment disregarded whether the patients who were sent messages opted-out or failed to receive the messages due to phone number change or technical difficulties.

1. “Exposed”: Patients who had a valid mobile phone number in their chart during the SMS program data capture process.
2. “Unexposed”: Patients who had a chart available for review but for whom a valid mobile phone number was not identified in the chart. This situation could be because they did not have, or did not choose to provide, a mobile phone number at the time of enrollment; rare instances of erroneous recording of the phone number were also noted.
3. “Unknown”: All remaining patients in the cohort, for whom the presence or absence of a mobile phone number was not determined due to missing charts. Common reasons for missing charts were use of the chart elsewhere in clinic or loss to follow-up.

Each patient was observed for a specific subset of the observation period, as determined by the following censoring criteria. The first day of the observation period was July 1, 2013. Within this period, observations began at the earliest of: 1) the day after the end of the patient’s first recorded prescription during the pharmacy data availability period (November 2012 through June 2014), or 2) the day of the patient’s second recorded prescription (to avoid including patients who had not clearly established care in the clinic). We right-censored patients on the earlier of their transfer-out date or June 26, 2014 (i.e., the last available day of pharmacy data). For baseline characteristics, continuous values were compared with ANOVA; categorical values were compared with the Chi-squared test.

Our primary outcome was ART prescription coverage. A day was defined as covered if the patient had valid prescriptions that, if filled and taken as prescribed, allowed the patient to possess sufficient medication for the intended three-drug ART regimen on that day; otherwise, the day was considered not covered. Each day during the study period was a unit of observation, and thus each patient was weighted by their length of observation. The rates of patients achieving > 95% coverage before versus during the SMS program were compared using the one-sample McNemar’s test.

The daily unit of observation was chosen because: 1) several covariates and the outcome varied through time, and thus averaging these values across extended time periods would obscure the temporal relationship between covariates and the outcome; and 2) averages across longer time periods were heavily skewed to the right and thus could not be adequately modeled by standard distributions. If a patient had days of prescription coverage remaining at the time of a new prescription, the remaining days of coverage were carried over to the next prescription, up to a maximum of 30 days per clinic policy, if there was no change in the antiretrovirals. For example, if a patient had 15 days of prescription coverage remaining, and came early to an appointment at which they received a 30-day prescription for the same antiretrovirals, they would have 45 days of prescription coverage available. Any records of prescriptions that simply re-recorded the remaining balance of a 6-month prescription (e.g., a 3-month prescription recorded 3 months after a 6-month prescription) were treated as duplicates.

To account for repeated measures within an individual, we used mixed effects multivariable logistic regression models. No univariable results were determined; our *a priori* interest was in the adjusted results as described below. All models were fit using Stata version 13.1 (StataCorp, College Station, TX, USA).

In the regression model, we determined if SMS program exposure was associated with ART prescription coverage. Fixed effects were study group (i.e., Exposed, Unexposed, and Unknown), time period (i.e., pre-program versus during program), and factors available in the database that have been shown to influence adherence. Specifically, these factors were age [21], gender [21], prior duration of ART [21], prescription duration, first or second-line ART regimen [22], and fixed-dosed combination ART [23]. The “pre-program period” was defined as the two months prior to the start of the SMS program (i.e., July 1 through September 9, 2013), which served as a baseline level of adherence. We used an interaction term between study group and time period to determine the effect size of exposure to the SMS program. The model included a random intercept at the patient level. We used stepwise backward elimination (threshold *P* = 0.25) [24], with the program groups, time period, and their interactions retained *a priori*, to select the covariates for the executed model.

To estimate the absolute risk reduction of SMS program, the model calculated the time-averaged prescription coverage for the Exposed patients in the during-program period. We then applied the odds ratio of coverage given non-exposure during the program (comparing the Unexposed and Exposed groups). This calculation yielded the hypothetical coverage that an average patient would have had if they had not been exposed to the SMS program. The difference between the actual coverage and the hypothetical non-exposure coverage equaled the absolute risk reduction for a patient with average coverage.

Given the WHO emphasis on providing differentiated care [18] as well as the possibility that SMS interventions would need to be targeted to at-risk populations to achieve cost-effectiveness, we examined two subsets of patients using the same method as that used for the full cohort.

1. The <100% baseline coverage subset: patients with pre-program prescription coverage < 100%, suggesting at least one missed prescription during that period.
2. Recent ART initiators subset: patients who initiated ART within 2 years of the start of the SMS program, because such patients have an accelerated rate of attrition compared to patients with longer treatment histories [25].

## Results

### Full cohort

#### Patient characteristics

A total of 2,920 patients received ART during the pharmacy data availability period of November 4, 2012 to June 26, 2014. Of these, 665 did not meet our inclusion criteria for the following reasons: age <18 years (*N* = 458), no ART prescription in the observation period (*N* = 33), not enrolled at Ethembeni prior to the observation period (*N* = 160), delayed capture of phone number into the SMS program (*N* = 1), and no observation time (e.g., patient transferred out before end of first prescription) (*N* = 13). The study cohort therefore consisted of 2,255 patients: 1,771 (78.5%) in the Exposed group, 316 (14.0%) in the Unexposed group, and 168 (7.5%) in the Unknown group. The introductory SMS was sent to all 1,771 patients in the Exposed group. Among these patients, 1,287 (72.7%) had at least one confirmed successful SMS delivery (during the time this data were available, January 13 through June 26, 2014) and among these, 1,044 (81.1%) remained subscribed to the program for their entire time of observation.

Patients included in the full cohort analysis were similar to the general clinic population in terms of age and gender (Table 1). The full cohort Exposed and Unexposed groups were similar except that the Exposed group was younger and had a greater proportion of females compared to the Unexposed group. The Unknown group significantly differed from the Exposed and Unexposed groups in terms of several variables. Additionally, missing data was higher in the Unknown group compared to the Exposed and Unexposed groups. Of note, rates of fixed dose combination use increased for all groups during the observation as part of a nationwide rollout of the formulation.

**Table 1:** Characteristics of patients included in the retrospective full cohort

Variable	Exposed (*n* = 1771)	Unexposed (*n* = 316)	Unknown (*n* = 168)	*P*-value for all	*P*-value for Exposed vs. Unexposed
Age in years (IQR)	**37.7 (32.4–43.9)**	**38.9 (33.1–46.6)**	**37.7 (31.7–42.9)**	**0.015**	**0.006**
Female	**1,253 (70.8%)**	**203 (64.2%)**	117 (69.6%)	0.068	**0.020**
ART duration in years (IQR)	2.4 (1.5–3.3)	2.5 (1.6–3.5)	2.5 (1.2–3.4)	0.270	0.120
Prescription length in days (IQR)	**180 (65–180)**	**180 (66–180)**	**90 (36–180)**	**<0.001**	0.846
Patients (%) with…				**<0.001**	0.710
1-month prescriptions	**227 (12.8%)**	**43 (13.6%)**	**48 (28.6%)**		
2-month prescriptions	**282 (15.9%)**	**48 (15.2%)**	**24 (14.3%)**		
3-month prescriptions	**234 (13.2%)**	**35 (11.1%)**	**18 (10.7%)**		
6-month prescriptions	**1028 (58.0%)**	**190 (60.1%)**	**78 (46.4%)**		
First line regimen	**1,675 (94.6%)**	**295 (93.4%)**	**151 (89.9%)**	**0.041**	0.383
Fixed dose combination	**28 (1.6%)**	**5 (1.6%)**	**9 (5.4%)**	**0.002**	0.999
No pre-program observation time	**93 (5.3%)**	**22 (7.0%)**	**25 (14.9%)**	**<0.001**	0.220
Transfer out during observation	61 (3.4%)	8 (2.5%)	9 (5.4%)	0.269	0.507

*IQR* interquartile range

Values reflect data collection on November 4, 2012 (for age and ART duration) or as of first statistical observation (for all others). Bold indicates statistical significance (*P* < =0.05)

### Prescription coverage for the full cohort

**Fig. 2:**
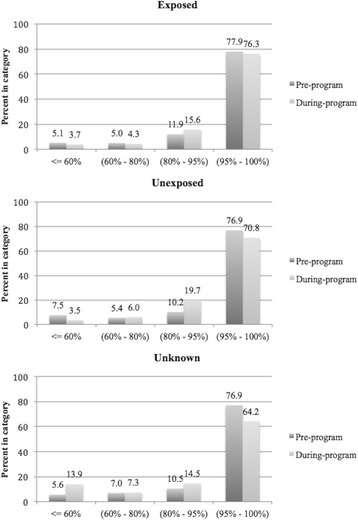
Distribution of per-patient prescription coverage in the full cohort. Pre-program refers to the two months prior to the start of the SMS program

The distribution of prescription coverage by patient before and during the program period is shown in Fig. 2. Prescription coverage was right-skewed, with the majority of patients achieving >95% coverage in both periods. For all three groups, this proportion of patients with coverage >95% decreased from the pre- to during-program period; this decrease was 2% (*P* = 0.277) for the Exposed group, compared with 6% (*P* = 0.118) and 13% (*P* = 0.038) for the Unexposed and Unknown groups, respectively.

### Effect of SMS program exposure on prescription coverage in the full cohort

After adjusting for age, gender, prescription lengths, regimen type, fixed dose combination, and pre-program prescription coverage, patients in the Exposed group had greater odds of daily prescription coverage during the SMS program (AOR 1.23, 95% CI: 1.13–1.34, P < 0.001) compared to those in the Unexposed group. For an Exposed patient at an average level of adherence (94.1% during the program), the absolute risk reduction of exposure to the SMS program was 4.7 days of ART prescription coverage missed per year compared to the Unexposed group.

Full cohort patients in the Unknown group (compared to those in the Unexposed group) had higher odds of pre-program coverage (AOR 1.60, 95% CI: 1.022–2.51, *P* = 0.040), but after adjustment for this factor, had lower odds of coverage during the program (AOR 0.47, 95% CI: 0.41–0.54, *P* < 0.001). As shown in Table 2, increasing age, female gender, longer prescription length, use of a first line regimen, and use of a fixed dose combination were also associated with increased prescription coverage in the full cohort.

**Table 2:** Pre- versus during-SMS program differences in prescription coverage

	Full Cohort,*N* = 2255	<100% Baseline Coverage Subset, *N* = 725	Recent ART Initiators Subset, *N* = 416
Covariate	Adjusted Odds Ratio (95% CI); *P*-value		
Age (per year)	**1.03 (1.01–1.04);** ***P*** **< 0.001**	**1.02 (1.01–1.03);** ***P*** **= 0.001**	**1.03 (1.01–1.06);** ***P*** **= 0.016**
Female gender	**1.55 (1.26–1.90);** ***P*** **< 0.001**	**1.32 (1.03–1.68);** ***P*** **= 0.027**	0.92 (0.54–1.56); *P* = 0.751
Prior ART duration (per 30 days)	**–**	**–**	**–**
Prescription length (per 30 days)	**1.28 (1.27–1.29);** ***P*** **< 0.001**	**1.17 (1.16–1.19);** ***P*** **< 0.001**	**1.31 (1.28–1.34);** ***P*** **< 0.001**
First line regimen	**2.70 (2.33–3.12);** ***P*** **<0.001**	**4.30 (3.62–5.11);** ***P*** **< 0.001**	**5.52 (3.92–7.77);** ***P*** **< 0.001**
Fixed dose combination	**2.71 (2.62–2.81);** ***P*** **< 0.001**	**3.51 (3.33–3.70);** ***P*** **< 0.001**	**2.88 (2.67–3.10);** ***P*** **< 0.001**
During-program period	**0.61 (0.56–0.66);** ***P*** **< 0.001**	**1.56 (1.42–1.71);** ***P*** **< 0.001**	**0.53 (0.45–0.63);** ***P*** **< 0.001**
Pre-program period coverage (by Group):			
Exposed vs. Unexposed	1.05 (0.79–1.39); *P* = 0.732	1.00 (0.72–1.39); *P* = 0.992	**2.53 (1.11–5.77);** ***P*** **= 0.028**
Unknown vs. Unexposed	**1.60 (1.02–2.51);** ***P*** **= 0.040**	1.48 (0.85–2.57); *P* = 0.166	**3.07 (1.01–9.35);** ***P*** **= 0.049**
Program Effect (= During-program period coverage, by Group, adjusting for the variables above):			
Exposed vs. Unexposed	**1.23 (1.13–1.34);** ***P*** **< 0.001**	**1.29 (1.17–1.43);** ***P*** **< 0.001**	1.17 (0.97–1.42); *P* = 0.101
Unknown vs. Unexposed	**0.47 (0.41–0.54);** ***P*** **< 0.001**	**0.67 (0.57–0.80);** ***P*** **< 0.001**	**0.33 (0.24–0.45);** ***P*** **< 0.001**

This table presents output from the mixed effects logistic regression on prescription coverage for a given day. The *Groups* are as follows: “Exposed” patients were sent the SMS; “Unexposed” patients did not have mobile phone numbers and thus were not sent SMS; “Unknown” patients had unknown phone number statuses due to missing charts, and thus also were not sent SMS. The “<100% Baseline Coverage Subset” includes patients with coverage < 100% in the pre-program period. The “Recent ART Initiators Subset” includes patients who initiated ART within 2 years of the SMS program start. The backward stepwise selection process of model parameters led to the omission of prior ART duration. *CI* = confidence interval. Bold indicates statistical significance at *P* < =0.05

### Subset analyses

The subset of patients with <100% baseline coverage consisted of 576, 104, and 45 patients for the Exposed, Unexposed, and Unknown groups, respectively. The baseline characteristics for the Exposed and Unexposed subgroups did not demonstrate any statistically significant differences; the Unknown subgroup differed from the others in having a lower prevalence of first line regimen use at study baseline (84.4% compared to 95.0 and 92.3% for the Exposed and Unexposed, respectively) and a higher proportion of missing data (8.9% compared to 0.4 and 1.0% for the Exposed and Unexposed, respectively). The subset of patients who initiated ART within two years of the SMS program start date consisted of 333, 41, and 42 patients for the Exposed, Unexposed, and Unknown groups, respectively. There were no statistically significant differences in the baseline covariates among these groups (data not shown).

For the <100% baseline coverage subset, the SMS program was associated with an increase in prescription coverage when comparing the Exposed and Unexposed subgroups (AOR 1.29, 95% CI: 1.17–1.43, P < 0.001). The corresponding absolute risk reduction of exposure to the SMS program for an Exposed patient with average coverage in this subset was 8.2 days of ART prescription coverage missed per year compared to the Unexposed group. For the recent ART initiator subset, no statistically significant differences were seen in prescription coverage between the Exposed and Unexposed subgroups (AOR 1.17, 95% CI: 0.97–1.42, *P* = 0.101). As shown in Table 2, increasing age, longer prescription length, use of a first line regimen, and use of a fixed dose combination were also associated with increased prescription coverage in both subsets.

## Discussion

This study assessed a weekly SMS program implemented by a government-run clinic in rural South Africa with limited outside funding, no incentives to encourage participation, and no dedicated research staff, setting it apart from prior research studies reported in the literature. Using routinely collected pharmacy data, we found that a weekly SMS program had a positive effect on ART adherence in a real world setting; however, the magnitude of this effect was small (average of 4.7 days per year). The effect for an average patient with <100% baseline prescription coverage was 8.2 days per year, but there was no significant effect for patients who had initiated ART within two years of the SMS program, possibly due to the relatively small size of the subset.

The clinical significance of the measured benefit of the SMS program among the full cohort is unclear. Although CD4 counts and viral loads were not consistently available for comparison in this cohort, prescription coverage has been shown to predict virologic suppression [26]. Prescription coverage is conceptually the ceiling on pill-taking adherence, the true value of which may be lower than the measured prescription coverage. Moreover, both the pattern and average level of adherence impact clinical outcomes. Despite the relatively long half-life of efavirenz, which is in the first-line regimen in South Africa, increasing duration of treatment interruption, even on the order of a few days, can increase risk of viral rebound [27, 28].

Many of the recorded lapses in prescription coverage may represent treatment interruptions, highlighting the potential clinical significance of the measured program effect. Future study of the downstream effects of real-world SMS programs on viral suppression and other clinical outcomes will help clarify the broader public health impact of such programs.

The exact mechanism of the improvement in prescription coverage is not known, but may reflect perceptions of social support and encouragement to remain in care. SMS programs have generally been well-accepted, including this one as described in a separate report [20]. Other studies involving qualitative assessments of SMS adherence interventions have noted a sense of social support through the messages [7, 29]. Compared to that of the full cohort, the SMS program effect size was possibly stronger among patients in the <100% baseline coverage subset, who likely had missed at least one prescription and/or appointment during their pre-program period. Missed appointments have been found to initiate a cascade of disengagement from care [5], and the SMS program’s mechanism could partially be via interruption of this process. Notably, the SMS program did not show a significant positive benefit among the recent ART initiators subset, suggesting that the intervention’s effect is not primarily via helping new patients to establish care.

The Unknown group (approximately 7% of the cohort) had a disproportionate share of patients with adherence challenges. It included patients whose charts were outside the file room during the period of data capture, which generally indicated the presence of clinical challenges or being lost to follow-up. Given these aspects of the Unknown group, we do not believe it is an unbiased comparator to the Exposed group. These issues also emphasize the importance of capturing phone numbers at enrollment for all individuals in care to help to ensure all vulnerable patients are offered the program.

While this study was not a randomized controlled trial, we believe the Unexposed group is a reasonable comparator to the Exposed group and adds value in assessing the impact of the SMS program on ART adherence. One potential source of confounding is differential phone ownership in the Unexposed group, which was not assessed at the time of the SMS program implementation. Rather, patients were asked for their contact phone numbers at clinic enrollment, which was on average 3.3 years prior to SMS program launch. However, we believe this possibility was unlikely to confound our results because, during this time, the prevalence of mobile phone ownership among South African adults was increasing and reached 90% in 2013 [30]. Thus, we anticipate most of the patients in the Unexposed group had mobile phones, but they were not known to the clinic. We also believe patients were unlikely to have withheld their phone numbers as another study from an HIV clinic in the region (albeit one that charged state-subsidized clinic fees) found that 99% of individuals with cell phones were willing to be contacted by the clinic on these phones [31]. That study also showed that age and employment were socio-economic factors associated with mobile phone ownership [31]. In our study, we were able to control for age. While we could not directly control for employment, we used the pre-program prescription coverage and patient-level random intercept to adjust for confounders of the effect of the SMS program on ART prescription coverage.

This analysis has other limitations. First, the unit of observation of each day was powered to detect small effects. We addressed the repeated measures aspect of this approach by using a mixed model. Second, the prescription lengths were long (up to 6 months) relative to the observation time (up to about 12 months), thus limiting our ability to identify lapses in prescription coverage. Third, any errors in clinical data collection may have incorrectly identified patients as non-covered, including patients who transferred out but did not receive the appropriate documentation from the clinic.

## Conclusions

We found that exposure to the clinical SMS program had a small measurable clinical impact among the full cohort that was possibly stronger among patients with imperfect pre-program prescription coverage. Generalizability to similar government ART clinics in the region is likely high.

We believe three primary avenues of future work should be pursued. First, additional studies should further explore the mechanisms by which SMS programs affect adherence, particularly their effects on social support [7, 29]. Second, long-term follow-up studies on the durability of impact would be informative for the return on investment in this type of intervention. Third, additional cohort studies of other clinical SMS programs should assess the reproducibility of the results in routine clinical settings.

In light of the high overall adherence, the cost-effectiveness of such implementations could be enhanced by improving their designs and targeting enrollment to individuals at risk for adherence challenges.

## Abbreviations

AOR: Adjusted odds ratio
ART: Antiretroviral therapy
CI: Confidence interval
SMS: Short message service
WHO: World Health Organization
